# Rehabilitation Outcomes: Ischemic versus Hemorrhagic Strokes

**DOI:** 10.1155/2015/891651

**Published:** 2015-07-12

**Authors:** Robert Perna, Jessica Temple

**Affiliations:** The Institute for Rehabilitation and Research, Houston, TX 77073, USA

## Abstract

*Background*. Ischemic and hemorrhagic strokes have different pathophysiologies and possibly different long-term cerebral and functional implications. Hemorrhagic strokes expose the brain to irritating effects of blood and ischemic strokes reflect localized or diffuse cerebral vascular pathology. *Methods*. Participants were individuals who suffered either an ischemic (*n* = 172) or hemorrhagic stroke (*n* = 112) within the past six months and were involved in a postacute neurorehabilitation program. Participants completed three months of postacute neurorehabilitation and the Mayo Portland Adaptability Inventory-4 (MPAI-4) at admission and discharge. Admission MPAI-4 scores and level of functioning were comparable. *Results*. Group ANOVA comparisons show no significant group differences at admission or discharge or difference in change scores. Both groups showed considerably reduced levels of productivity/employment after discharge as compared to preinjury levels. *Conclusions*. Though the pathophysiology of these types of strokes is different, both ultimately result in ischemic injuries, possibly accounting for lack of findings of differences between groups. In the present study, participants in both groups experienced similar functional levels across all three MPAI-4 domains both at admission and discharge. Limitations of this study include a highly educated sample and few outcome measures.

## 1. Introduction 

Stroke, whether ischemic or hemorrhagic in nature, has the ability to culminate in devastating clinical outcomes. Stroke is the fourth leading cause of death in the United States, with 795,000 people suffering from stroke every year (“Stroke Statistics,” n.d. [1]). Of those, 600,000 are first attacks and 185,000 are recurrent attacks, with more than 140,000 people dying from a stroke each year [1].

Strokes can be broadly classified as hemorrhagic or nonhemorrhagic. Intracerebral hemorrhage (ICH) encompasses 10% to 15% of all strokes [2–4]. ICH occurs from rupture of cerebral vessels, often as the result of high blood pressure exerting excessive pressure on arterial walls already damaged by atherosclerosis, aneurysm, or arteriovenous malformation [5]. Ischemic strokes or cerebral infarcts (CI) are the result of development of thrombi and/or emboli leading to blockages and lead to deficiency of oxygen in vital tissues [6]. Decreased and/or absent cerebral circulation causes neuronal cellular injury, inflammatory responses, and neuronal death [6]. Each stroke subtype (hemorrhagic versus nonhemorrhagic) can be subdivided. For example, ICH can be subdivided into primary and secondary ICH. Primary ICH, comprising 78% to 88% of all hemorrhages, derives from the spontaneous rupture of small vessels damaged by chronic hypertension or amyloid angiopathy [4, 5]. Secondary ICH results from bleeding of cerebrovascular vascular abnormalities, tumors, or impaired coagulation [5].

ICH is associated with a higher risk of fatality compared with cerebral infarction and approximately half of all patients with primary ICH die within the first month after the acute event [2–4, 7]. Additionally, patients who are aged 85 and above, compared to younger patients, tend to experience higher clinical severity (moderate or severe neurological deficit at time of hospital discharge of 89% versus 58%) and greater in-hospital mortality rate (50% versus 27% [8]). Those who suffer ischemic strokes have a much better chance for survival than those who experience hemorrhagic strokes, as hemorrhagic stroke not only damages brain cells but also may lead to increased pressure on the brain or spasms in the blood vessels [9]. Of note, there are three main processes implicated in neurorecovery: angiogenesis, neurogenesis, and synaptic plasticity. These processes are naturally produced in adult brains subsequent to intensive rehabilitation, which could promote an endogen neurorepair phenomenon [10].

Prior research has uncovered numerous predictors of poor functional outcome, including bowel and urinary incontinence, longer interval between the onset of stroke and hospital admission, more severe hemiparesis upon admission, visuospatial deficits, and lower FIM admission score [11]. Additionally, for ischemic stroke, worse initial stroke severity, older age, being female, prior history of stroke, initial neurologic deficit, lesion location, diabetes mellitus, high fever within three days after the stroke, and neurological complications are related to poorer functional outcome after stroke [12–15]. The relation to lesion location has been inconsistently reported to impact recovery, some research showing that stroke recovery was worse with deep subcortical strokes rather than superficial cortical areas and other studies showing the opposite outcome [16]. Additionally, those who experienced lacunar infarcts tended to have a better functional prognosis [17]. Those who were admitted to the hospital within 30 days of their stroke were both admitted and discharged with higher functional scores than those admitted after 30 days, and the length of stay was significantly shorter [18]. Moreover, higher admission Functional Independence Measure (FIM) score was associated with higher probability of functional improvement during rehabilitation [19].

Scant research has examined the functional outcome differences in those with hemorrhagic versus ischemic strokes. Although it is generally believed that hemorrhagic stroke survivors have better neurological and functional prognoses than nonhemorrhagic stroke survivors, data are mixed. On one hand, one study found that although there were no differences in discharge FIM or FIM gain between stroke types, hemorrhagic cardiovascular accident (CVA) patients showed somewhat faster functional motor gains and had shorter length of stay but did not exhibit faster cognitive gains [20]. However, the generalizability of these data was limited because of the use of few outcome variables and the small size of the sample. Conversely, one study found that those with ICH exhibited slower but greater recovery than those with CI [19]. Paolucci et al. [21] found that those with hemorrhagic CVAs had higher Canadian Neurological Scale and Rivermead Mobility Index scores at discharge, higher effectiveness and efficiency, and a probability of a high therapeutic dose that was 2.5 times greater than those with ischemic CVAs. One study found that those with ICH exhibited greater admission functional impairment, although no difference in total discharge FIM score was present between those with ischemic and hemorrhagic CVA [22]. Additionally, younger age, longer length of stay, and admission FIM cognitive score predicted total discharge FIM score recovery [22].

On the other hand, one study demonstrated that, among conscious stroke patients, ICH predicted poor neurologic outcome, nearly doubling the odds of long-term disability as compared to ischemic stroke [23]. However, some studies show no functional differences between the two groups. For example, Franke et al. [24] observed no difference in level of functional independence after one year of follow-up between ICH and CI patients and concluded that the extent of the brain lesion was the determining factor in outcome in those who survive the first two days after CVA.

Stroke severity appears to be an influential factor in predicting outcome. In one study, stroke type had no influence on mortality, neurological or functional outcome, or time course of recovery, with initial stroke severity, the most important factor [25]. The authors concluded that poorer prognosis in those with ICH is due to the increased frequency of those ICH who experienced increased stroke severity [25]. Similarly, in another study, those with more severe ICH exhibited significantly greater recovery than those with CI of a similar CVA severity [22].

The aim of the present study was to determine if there are any differences in functional recovery between those with ICH and CI CVAs. As the majority of the research conducted on inpatients who experienced CVAs showed that those with ICH CVAs exhibited more functional recovery, in the present study, it was hypothesized that individuals with hemorrhagic strokes would show more functional recovery by completion of postacute brain injury rehabilitation than individuals with ischemic strokes.

## 2. Methods

### 2.1. Participants

This was a retrospective study which utilized archival data. Participants were comprised of 284 outpatients at a Southwestern treatment facility (ischemic = 172, hemorrhagic = 112) who were diagnosed with CVAs. Although it would be interesting to know the frequency of lacunar versus nonlacunar ischemic strokes in the present study, those data were unavailable. Nearly all participants were within six months after stroke. Specific length of time between stroke and admission to rehabilitation was largely in the first three months after stroke. All participants were consecutive referrals for treatment early after discharge from an inpatient setting and the only inclusion criteria were completion of the program. The demographic and clinical characteristics of the final study sample are shown in Tables 1 and 2, respectively. The mean age of those with ischemic CVAs was 56.08 years and of those with hemorrhagic CVAs was 54.30 years. The mean level of education for those with ischemic CVAs was 16.48 and for those with hemorrhagic CVAs was 16.6. The majority of those with ischemic and hemorrhagic CVAs were male. The majority of those with both ischemic and hemorrhagic CVAs were identified as Caucasian or African American, with a smaller percentage being Asian, Hispanic, or unspecified. Using ANOVA, there were no differences between groups in terms of age (*F*(3, 349) = 0.571,* p* > 0.05), years of education (*F*(3, 345) = 0.925,* p* > 0.05), race (*χ²*(15) = 22.149,* p* > 0.05), or gender (*χ²*(3) = 1.093,* p* > 0.05). Upon admission, both those with ischemic and hemorrhagic CVAs were reported to have mild to moderate limitations in terms of physical and cognitive abilities, community integration and productivity, and overall functioning, and mild limitations in terms of psychosocial functioning. Upon discharge, both those with ischemic and hemorrhagic CVAs were reported to have mild limitations in terms of physical and cognitive abilities, community integration and productivity, psychosocial functioning, and overall functioning.

### 2.2. Measure

*The Mayo Portland Adaptive Inventory-4 (MPAI-4 [26])*. The MPAI-4 is a 35-item measure that assesses disability after brain injury, including impairment in the areas of physical, cognitive, emotional, behavioral, and social functioning. Items are scored on a 5-point Likert scale ranging from zero (no functional disabilities on that item) to four (indicating interference with activities more than 75% of the time). The measure has three subscales: Ability Index (ranging from 0 to 47), Adjustment Index (ranging from 0 to 46), and Participation Index (ranging from 0 to 30) and yields an overall score of 0–111, with lower scores indicating greater functioning. The Abilities Index is comprised of 12 items and evaluates abilities such as mobility and memory. The Adjustment Index utilizes nine items and assesses emotional and behavioral symptoms such as depression and fatigue. The Participation Index includes items measuring aspects of independence. Test-retest reliability ranged from adequate to excellent in children with acquired brain injury [27]. Internal consistency was 0.89 for the total score and ranged from 0.76 to 0.83 for the subscales [28]. There was good predictive validity for posttreatment outcome, level of functioning, return to employment, and independent living status [29, 30]. There was good discriminant validity among TBI groups [31].

### 2.3. Procedures

All participants were involved in a postacute outpatient program that included twice weekly treatment for three months, comprised of occupational therapy, speech therapy, physical therapy, social work, and neuropsychological services. The composition of care was determined based on intake evaluations of stroke symptoms upon admission. Each week, participants received one hour of neuropsychology services and one hour of social work services, whereas physical and occupational therapy varied from 2 to 4 hours per week. Each participant was rated by staff on the MPAI-4 shortly after the admission assessment and again prior to discharge. MPAI-4 change scores (ΔMPAI-4) were created by subtracting admission from discharge scores. Between groups ANOVAs were run for ΔMPAI-4 scores across all MPAI-4 dimensions. Additionally their productivity (return to work, school, and other activities) was rated at admission and discharge.

### 2.4. Statistical Analysis

All of the measures and data were assessed for normality assumptions and were found to be normally distributed. Repeated measures ANOVAs and one-way ANOVAs were utilized to determine the differences between groups at admission and discharge as well as determine the differences in change scores on total score, cognitive/physical abilities, participation, and psychosocial adjustment. Chi square was used to compare nominal level variables. Linear regression was also utilized to determine the extent to which type of stroke accounted for the variance in outcomes.

## 3. Results

Linear regression examining type of stroke (ischemic versus hemorrhagic) as a predictive factor of change in overall functioning was not significant (*F*(1, 165) = 1.778,* p* > 0.05) and explained 1.1% of the variance. Between groups ANOVA comparisons showed no significant group differences upon admission in terms of cognitive/physical abilities (*F*(3, 160) = 2.145,* p* > 0.05), participation (*F*(3, 158) = 1.122,* p* = 0.342), psychosocial adjustment (*F*(3, 210) = 0.044,* p* > 0.05), or overall functioning (*F*(3, 165) = 1.069,* p *> 0.05). There were no significant group differences at discharge in terms of cognitive physical abilities (*F*(3, 160) = 0.88,* p >* 0.05), adjustment (*F*(3, 209) = 0.061,* p >* 0.05), participation (*F*(3, 162) = 0.251,* p* > 0.05), or overall functioning (*F*(3, 167) = 0.431,* p* > 0.05). Additionally, there were no significant differences in change scores from admission to discharge regarding adjustment (*F*(3, 205) = 0.109,* p* > 0.05), cognitive/physical abilities (*F*(3, 154) = 1.563,* p* > 0.05), participation (*F*(3, 156) = 2.086,* p* > 0.05), or overall functioning (*F*(3, 163) = 1.138,* p* > 0.05).

Both groups (ischemic/hemorrhagic) had a very high level of employment before injury (71% and 85.7%, resp.), a very low level of employment at the onset of postacute rehabilitation (3.5% and 8.9%, resp.), but considerably improved employment at time of discharge (22% and 23.2%, resp.; see Tables 3 and 4).

## 4. Discussion

Overall, though the pathophysiology of these conditions is very different, both groups exhibited similar functional levels across all three MPAI-4 domains both at admission and discharge. Both groups showed improvements in functioning during rehabilitation, progressing from mild-moderate limitations to mild limitations across domains. Both groups exhibited a similar level of functional gains from treatment (admission to discharge). This suggests that both CVA types benefit equally from the same length and types of rehabilitation.

The data did not support the research hypothesis, as none of the scores (admission, discharge, or change scores) differed significantly between stroke subtypes. Both groups entered the program with a similar level of impairment as measured by the MPAI-4 and were discharged at a similar level of impairment. It may be that these groups recover in similar manner. However, there may have been other variables which contribute to our findings. The participants generally had a high level of education. One study found that lower level of education influenced functional dependence in ischemic stroke survivors [32]. Therefore, the higher level of education in the present sample may partially positively and perhaps differentially influence group outcomes. This also may have limited the possibility of uncovering differences among the groups, due to higher level of premorbid functional status and cognitive reserve. Moreover, the present study was comprised of a relatively young sample. Most strokes (approximately 75%) occur in individuals over the age of 65, significantly older than the present sample [1]. In some studies, age was found to be a predictor of functional outcome after rehabilitation, particularly regarding activities of daily living [22, 33]. Therefore, in the present study, both individuals who sustained ICH and CI CVAs might have exhibited better outcome due to younger age at time of stroke. Also, the participants in the present study were comprised of diverse ethnic groups. Some studies have found that Caucasian non-Hispanic patients exhibited higher postacute admission and discharge functional status ratings compared with patients in minority groups, particularly with older age [34]. Similarly, in another study, African American patients achieved less functional improvement at discharge from an inpatient rehabilitation facility [35]. The diverse ethnic sample in the present study may have made it difficult to uncover true functional differences among the participants.

The results of the present study were inconsistent with the literature. Several studies found differences in functional outcome between those with ischemic versus hemorrhagic CVAs; some studies found better functional prognosis in survivors with hemorrhagic CVA after inpatient rehabilitation [20, 21], whereas others found that those with ICH strokes exhibited greater functional impairment and greater improvement than ischemic strokes but progressed more slowly [19, 22]. Additionally, patients with hemorrhagic CVAs have a higher early mortality rate [2]. Therefore, those in the present study with hemorrhagic CVAs may not be truly representative of the general population. The results of the present study may differ from previous studies due to younger age, greater level of education, increased racial diversity, and high level of functioning upon admission to the postacute rehabilitation program.

There were several strengths to the present study. The types of treatments and number of treatment sessions per week per participant were relatively similar. Moreover, ratings of functional gain were completed by trained staff who worked closely with the participants over the three months of rehabilitation, allowing for a more accurate picture of functional ability. The study also used a widely utilized measure of functional outcome. Scant research has examined the difference in admission, discharge, and change scores regarding functional ability between subtypes of stroke. Additionally, the present study examined participants subsequent to outpatient rehabilitation, which, to our knowledge, has not been done with this population.

There were also several limitations to the present study. The admission scores were generally within the mild-moderate limitations range, limiting the possible range of scores. Additionally, the group was comprised of highly educated, young, and ethnically diverse individuals and therefore may not be generalizable to the general stroke population. The study only utilized one outcome measure, leading to the possibility of a limited depiction of functional outcome. Moreover, the study only utilized staff ratings and did not include patient and significant other ratings as supplementary information, which might have allowed for a fuller picture of functional outcome.

Future research should utilize additional outcome measures and more diverse measures when comparing these populations. It would be helpful to compare the populations from the initial admission into an inpatient unit with ratings three months after discharge from a postacute rehabilitation facility in order to better compare functional ability across time and length of rehabilitation. It might be useful to compare pre-CVA and postrehabilitation functional ability, particularly in the areas of psychosocial functioning and community participation to account for possible lower functional ability. It would be helpful to compare those who underwent early versus delayed admission in functional outcome between groups with CI and ICH. Future studies should match for age, education, ethnicity, and stroke severity. Similarly, it would be important to replicate this study with a more heterogeneous sample to explore to what extent these findings are similar in a broader population of individuals with CVA. Finally, future researchers should examine social support systems in order to determine the impact of social support on functional outcome in the participants.

Regarding clinical implications, these results show that both groups benefit from the same type, length, and frequency of rehabilitation. Due to residual impairments after three months of rehabilitation, it is recommended that treatment be extended for another few months, either at the postacute or outpatient level.

## Figures and Tables

**Table 1:** Demographic characteristics of participants.

Variable	Ischemic	Hemorrhagic
Age	56.08 (SD = 12.45)	54.30 (SD = 12.03)
Years of education	16.48 (SD = 11.57)	16.6 (SD = 11.64)
Gender (male)	110 (64%)	78 (69.5%)
Race		
Caucasian	89 (51.7%)	64 (57.1%)
African American	60 (34.9%)	30 (26.8%)
Hispanic	12 (7.0%)	13 (11.6%)
Asian	8 (4.7%)	4 (3.6%)
Unspecified	3 (1.7%)	0 (0.0%)

**Table 2:** Clinical characteristics of participants.

Variable	Ischemic	*T*-scores	Hemorrhagic	*T*-scores
Admission abilities	17.12 (SD = 6.62)	48	18.78 (SD = 7.41)	49
Admission participation	18.22 (SD = 6.53)	47	19.09 (SD = 6.25)	48
Admission adjustment	10.79 (SD = 5.12)	39	10.96 (SD = 4.79)	39
Admission total	46.19 (SD = 15.59)	47	48.02 (SD = 17.31)	48
Discharge abilities	9.06 (SD = 5.54)	39	5.99 (SD = 4.64)	31
Discharge participation	10.35 (SD = 7.35)	39	10.33 (SD = 7.21)	39
Discharge adjustment	5.99 (SD = 4.64)	33	5.93 (SD = 4.17)	33
Discharge total	25.16 (SD = 15.27)	35	24.06 (SD = 14.51)	35
Change abilities	8.11 (SD = 5.56)	—	9.85 (SD = 6.50)	—
Change participation	7.88 (SD = 5.52)	—	8.71 (SD = 6.25)	—
Change adjustment	4.71 (SD = 3.76)	—	5.03 (SD = 4.25)	—
Change total	21.04 (SD = 13.24)	—	23.95 (SD = 15.67)	—

**Table 3:** Productivity of participants with ischemic CVA.

Productivity	Preinjury	Admission	Discharge
Competitive employment	123 (71.5%)	6 (3.5%)	39 (22.7%)
Modified job	7 (4.1%)	9 (5.2%)	22 (12.8%)
School	3 (1.7%)	1 (0.6%)	6 (3.5%)
Homemaker	4 (2.3%)	5 (2.9%)	11 (6.4%)
Volunteer work	5 (2.9%)	2 (1.2%)	18 (10.5%)
Leisure	19 (11.0%)	24 (14.0%)	48 (27.9%)
Nonproductive	7 (4.1%)	125 (72.7%)	25 (14.5%)

**Table 4:** Productivity of participants with hemorrhagic CVA.

Productivity	Preinjury	Admission	Discharge
Competitive employment	96 (85.7%)	10 (8.9%)	26 (23.2%)
Modified job	1 (0.9%)	1 (0.9%)	8 (7.1%)
School	1 (0.9%)	0 (0.0%)	2 (1.8%)
Homemaker	2 (1.8%)	1 (0.9%)	4 (3.6%)
Volunteer work	2 (1.8%)	0 (0.0%)	12 (10.7%)
Leisure	6 (5.4%)	13 (11.6%)	29 (25.9%)
Nonproductive	3 (2.7%)	87 (77.7%)	29 (25.9%)
